# “A child’s nightmare. Mum comes and comforts her child.” Attachment evaluation as a guide in the assessment and treatment in a clinical case study

**DOI:** 10.3389/fpsyg.2014.00912

**Published:** 2014-08-20

**Authors:** Silvia Salcuni, Daniela Di Riso, Adriana Lis

**Affiliations:** Department of Developmental and Socialization Psychology, University of PadovaPadova, Italy

**Keywords:** assessment, attachment, psychodynamic supportive therapy, outcome research, clinical case study

## Abstract

There is a gap between proposed theoretical attachment theory frameworks, measures of attachment in the assessment phase and their relationship with changes in outcome after a psychodynamic oriented psychotherapy. Based on a clinical case study of a young woman with Panic Attack Disorder, this paper examined psychotherapy outcome findings comparing initial and post-treatment assessments, according to the mental functioning in *S* and *M*-axis of the psychodynamic diagnostic manual. Treatment planning and post-treatment changes were described with the main aim to illustrate from a clinical point of view why a psycho-dynamic approach, with specific attention to an “attachment theory stance,” was considered the treatment of choice for this patient. The Symptom Check List 90 Revised (SCL-90-R) and the Shedler–Westen Assessment Procedure (SWAP–200) were administered to detect patient’s symptomatic perception and clinician’s diagnostic points of view, respectively; the Adult Attachment Interview and the Adult Attachment Projective Picture System (AAP) were also administered as to pay attention to patient’s unconscious internal organization and changes in defense processes. A qualitative description of how the treatment unfolded was included. Findings highlight the important contribution of attachment theory in a 22-month psychodynamic psychotherapy framework, promoting resolution of patient’s symptoms and adjustment.

## INTRODUCTION

Attachment theory in Bowlby’s (1969/1982, 1973, 1980, 1988) and Ainsworth’s (1963, 1967) tradition postulates that an individual’s experience of early parental care contributes to the development of internal representations of self and others as safe and available. This theory offered the clinicians a scientific grounded model, which postulated and empirically demonstrated the origin of psychopathology in early separation experiences and in adverse emotional experiences (Oppenheim and Goldsmith, 2007; Cassidy and Shaver, 2008). The most recent literature endorses that attachment theory is consonant with all assessment and treatment approaches which evaluate childhood experiences as an important contributor to adult functioning (e.g., Wallis and Steele, 2001; Blatt and Levy, 2003; Diamond, 2004; Bakermans-Kranenburg et al., 2005; Buchheim et al., 2007; Zegers et al., 2008; Buchheim and George, 2011). Throughout the case formulation and the planning of treatment, attachment theory has also the potential to provide-at least-a useful foundation for defining the target of change in psychotherapy (e.g., features of internal working models or attachment patterns), understanding the processes through which change occurs (e.g., through the development of a secure base and exploration of working models; e.g., Fonagy, 1999, 2001; Cozzolino, 2002; Parish and Eagle, 2003; Mallinckrodt et al., 2005; Wallin, 2007; Fosha, 2009; Holmes, 2010; Siegel, 2010). As Bowlby originally stated, while reconsidering classical attachment theory, Davila and Levy 2006, p. 990) stressed *“five key tasks for psychotherapy: (a) establishing a secure base, which involves providing patients with a secure base from which they can explore the painful aspects of their life; (b) exploring past attachments, which involves helping patients explore past and present relationships, including their expectations, feelings, and behaviors; (c) exploring the therapeutic relationship, which involves helping the patient examine the relationship with the therapist and how it may relate to relationships or experiences outside of therapy; (d) linking past experiences to present ones, which involves encouraging awareness of how current relationship experiences may be related to past ones; and (e) revising internal working models, which involves helping patients to feel, think, and act in new ways that are unlike past relationship.”* Despite the increasing interest in the relevance of attachment theory as a framework to understand the unfolding of psychodynamic treatment, there is a gap between the proposed theoretical frameworks and the empirical measures of attachment used in the assessment, and only few studies addressed the interplay between attachment pattern measures, and their implication for unfolding and outcome in a psychoanalytic oriented treatment (Buchheim and Kachele, 2001; Dahlbender et al., 2004; Buchheim, 2005; Lis et al., 2008, 2011; Isaacs et al., 2009).

Interpersonal problems, adult attachment, and emotion regulation have been increasingly studied across adult anxiety disorders. Literature linked attachment and separation in infants and preschool children to separation anxiety disorder, agoraphobia, and panic attacks later in life, underlining how insecure attachment can lead to an increased risk for attachment psychopathology and subsequent social and emotional maladjustment/attachment and separation anxiety/school or work phobia/attachment correlations (Routh and Bernholtz, 1991). Of all the forms of anxiety, separation anxiety seems to be the one which is most likely to be associated with an anxious attachment style, because sufferers are by definition highly sensitive to real or perceived threats to relationships (Main et al., 1985). Separation anxiety would appear to be a core form of anxiety associated with panic attack disorder and with attachment problems (Hazan and Shaver, 1987; Bartholomew and Horowitz, 1991; Eng et al., 2001). Dysfunctional and not good-enough parenting and hereditary factors appear to play a role in generating early separation anxiety. However, the child’s anxiety itself may generate overprotective parenting (Manicavasagar et al., 1999, 2009) which, in turn, could make children approach their caregivers both in response to dangerous external stimuli and to caregiver’s permanent monitoring availability and attentiveness; moreover, overprotecting or over responsive parents could obstacle the expression of the explorative system, even when a “secure base” is provided (Pacchierotti et al., 2002). Although attachment theory suggests that anxious attachment styles are mostly associated with risks of developing anxiety disorders, neither all anxious attached patients develop panic attack disorder, nor all secure attached patients do not develop it: the latter is a weird and rare condition because, theoretically, secure early relationships with adults are the basis for the development of a sense of control and predictability accounting for normal subjects’ tendency not to interpret ambiguous internal stimuli as threatening (Shear, 1991).

Based on a clinical case study of a young woman with Panic Attack Disorder- Matilde-, this paper examined psychotherapy outcome findings comparing initial and post-treatment assessments, according to the mental functioning in *S* and *M*-axis of the* Psychodynamic Diagnostic Manual* (PDM; PDM Task Force, 2006)^1^. The patient’s choice is motivated by this “rare combination”: a PAD patient with secure attachment. The first aim of this paper was to provide incremental usefulness to the picture of the patient’s idiographic and intra-subjective features, using a multi- method assessment based on (1) two performance-based attachment measures – the Adult Attachment Interview (AAI; George et al., 1984/1985/1996), and The Adult Attachment Projective Picture System (AAP; George and West, 2001, 2012), (2) the Shedler–Westen Assessment Procedure (SWAP–200; Westen and Shedler, 1999a,b), and (3) a self-report symptom scale, the Symptom Checklist 90 Revised (SCL-90-R; Derogatis, 1983; Funder, 1997; Meyer et al., 1999; Ozer, 1999).The second aim was to describe how Matilde’s assessment findings – and more specifically attachment pattern analysis – could represent useful guidelines for the unfolding of a psychoanalytic therapy with a supportive approach, in an attachment theory framework (Misch, 2000).

We hypothesized that the AAI, the AAP, the SCL-90-R, and the SWAP–200 would help in focusing on the most relevant dimensions of patient’s psychological functioning which make a meaningful diagnosis (Barron, 1998; Shedler and Westen, 2007) at the beginning and at the end of treatment. Attention was directed to the interplay between modification of overt symptoms and behaviors, and changes in personality functioning and adaptation; more specifically, we focused on patterns and complexities in the patient’s internal organization and interpersonal functioning (Shectman and Harty, 1986; Peebles-Kleiger, 2002; Bram, 2010). A reduction in psychopathological symptoms and an improvement in mental functioning according to the PDM *M*-axis and *S*-axis were expected at the end of the therapy.

## CLINICAL CASE PRESENTATION: MATILDE

Matilde was a pleasant 20-year-old young woman, who looked younger than her age. She was a self-referred patient, and was assessed for a high level of anxiety at a university-based psychology-training clinic^2^. Matilde had a diagnosis of Panic Attack Disorder in Axis I (DSM-IV; American Psychiatric Association [APA], 1994), and no diagnosis in Axis II. Although she was a 2-year student at the Medical School with outstanding results, she felt “*anxious, confused, and insecure,*” “*I do not know if this Faculty is good for me, maybe Biology would be better, or Pharmacy … I do not know really, I am so confused; I do not understand what is happening to me …. I am no more sure about anything.*” Insecurity caused her quite severe crying crises, pervasive anxiety, and some physical symptoms, such as psychomotor agitation and tachycardia. She had taken light tranquilizers in the last 3 months. She felt unable to control or understand her present distress. Since she started University, her life had been totally busy with studying, leaving no time or desire to engage in social relationships. She did not talk about any actual satisfying relationships. The only “*friends*” she kept in touch with were schoolmates from high school, with whom she shared school topics. She had never had a boyfriend, and felt very uncomfortable talking about romantic or sexual topics. Matilde moved away from her small native town to attend University, and she was sharing an apartment with other students next to the Medical School. She went back home to her family during University vacations. She came from an intact family, which she was very proud of. She had a 10-year-old sister, Sarah, to whom she was very attached. Sarah was described as very different from Matilde: very funny, an ironic with a lot of energy. They spent a lot of time playing together, and Matilde was unconcerned about her worries when she was with Sarah. Matilde describes her childhood with some enjoyment and unconcern while her present appears very worrying, uncertain and without any source of protection and soothing. Matilde supports a good relation with her mother, although the father is described as rigid and very involved in practical duties.

## APPROACH TO THE CASE: PROCEDURE AND INSTRUMENTS

At the initial assessment phase Matilde underwent three interview sessions, two test sessions and one feedback session. In particular, Matilde’s evaluation involved the administration of the AAI and the AAP, the SCL-90-R, and the SWAP–200. All results were integrated with clinical interview contents to formulate a case conceptualization, according to specific dimensions of the PDM. In the feedback session, a once-a-week psychodynamic psychotherapy with a supportive approach was proposed to and accepted by Matilde. The therapy lasted 22 months. At treatment conclusion Matilde accepted to be re-administered the AAP and the SCL-90-R. Based on the last three sessions also the SWAP–200 was re-administered. All the tools administered were scored and interpreted by independent judges^3^. A brief description of used tools follows after timetable of administration (**Table 1**).

**Table 1:** Timetable of administered tools.

Administered∖time	T1	T2	T3	T4	T5
Patient with therapist	Three interview sessions	Two test session	Two feedback session	Therapy (lasted 22 months)	Follow-up assessment
Patient		SCL90R			SCL90R AAP
		AAI			AAP
		AAP			
Therapist		SWAP–200 PMD profile			SWAP–200 PMD profile

*Symptom Checklist 90 Revised* (Derogatis, 1983) is a 90-item self-report questionnaire scored on a five-point Likert scale of distress from 0 (none) to 4 (extreme), indicating the rate of occurrence of symptoms during the time reference (Derogatis et al., 1973). It is intended to measure symptom intensity on 10 different dimensions: somatization (SOM), obsessive–compulsive (O–C), interpersonal sensitivity (I-S), depression (DEP), anxiety (ANX, hostility (HOS), phobic anxiety (PHOB), paranoid ideation (PAR), psychoticism (PSY), and sleep difficulties (SLEEP). A Global Severity Index (GSI) of distress is calculated. According to the Italian Manual, an intensity raw score higher than one was qualified as penetrating in the clinical range. The internal consistency coefficient alphas for the nine symptom dimensions ranged from 0.77 for Psychoticism, to 0.90 for Depression. Test–retest reliability coefficients ranged between 0.80 and 0.90 after 1 week of therapy. The few validity studies of the SCL-90-R demonstrate levels of concurrent, convergent, discriminant, and construct validity comparable to other self-report inventories (Derogatis, 1983).

*The Shedler–Westen Assessment Procedure* (Westen and Shedler, 1999a,b) is a set of 200 personality-descriptive statements developed for clinicians to assess adult personality traits and pathologies (Shedler and Westen, 1998). Starting from clinical interviews, the assessor is asked to describe the patient by arranging the statements into eight categories, from those that are not descriptive (assigned a value of “0”) to those that are highly descriptive (assigned a value of “7”) for each of the 200 personality-descriptive variables. The instrument is based on the Q-sort method that requires clinicians to arrange items into a fixed distribution (Block, 1978). The SWAP–200 could be interpreted at a nomothetic as well as at an idiographic level. Nomothetic interpretations are carried out following two profiles. The first is the PD-T score profile of the 10 Personality Disorders included in DSM–IV (paranoid, schizoid, schizotypal, antisocial, borderline, histrionic, narcissistic, avoidant, dependent, obsessive); the Q-T profile covers 11 dimensions (psychological health, dysphoric, antisocial, schizoid, paranoid, obsessive, histrionic, narcissistic, avoidant, depressive high functioning, emotional dysregulation, dependent, hostile). Both PD-T and Q-T profiles include a score on a Healthy Functioning scale. Inter-rater reliability coefficients range from 0.70 to 0.80. Support for the validity of the SWAP–200 is derived from its ability to predict relevant variables in expected ways, including family psychiatric history, history of abuse, social, and school functioning, violence, suicidal behaviors and attempts, attachment status, and eating disorder diagnostic groups (Westen and Muderrisoglu, 2003a,b). Idiographic narrative case description is also included in the SWAP–200 (e.g., Lingiardi et al., 2006). Both levels were used to assess Matilde. Moreover, the SWAP–200 (Westen and Shedler, 1999a,b) is one of the instruments listed by PDM work-group members to be used to measure the dimensions of the *M*-axis.

*The Adult Attachment Interview* (George et al., 1984, 1985, 1996; Hesse, 2008) is an about 1 h audio-recorded semi-structured interview that explores an adult’s mental representations of attachment, guiding the individual through a series of questions about past and present relationships with each parent and attachment-relevant events during childhood. The AAI focuses on the assessment of the attachment internal working model (Bowlby, 1969/1982) and assumes developmental continuity of the attachment system along life. AAI final attachment classification is evaluated on two different set of scales (1) Experience Scales that evaluate for example Loving, Rejecting, Neglect, Role Reversal, Pressure to achieve and (2) State of Mind Scales that assess Coherence, Metacognitive Processes, Lack of Recall, Passivity of Discourse, Idealization, Anger, Derogation attitudes toward caregivers, Unresolved mourning or trauma, Feared loss of one’s own child. Starting from these scales, each interview is classified in one of the primary attachment patterns: secure/autonomous, dismissing/avoidant, and preoccupied/entangled or “cannot classify.” Where applicable, the “unresolved” pattern with respect to loss, trauma, or abuse could be scored. Multiple scoring is allowed (e.g., F/DS). AAI validation rests on more than 25 years of developmental and clinical research (van IJzendoorn and Bakermans-Kranenburg, 2008). Rigorous psychometric testing and meta-analyses of the AAI demonstrate its stability, and discriminant and predictive validity in both clinical and non-clinical populations. In a recent meta-analysis of 61 clinical samples (van IJzendoorn and Bakermans-Kranenburg, 2008), strong associations were found between psychiatric diagnoses (i.e., anxiety disorders, borderline personality disorder) and attachment insecurity.

*The Adult Attachment Projective Picture System* (George and West, 2001) is based on a standardized set of seven drawn pictures divided in Alone and Dyadic stimuli^4^. The pictures describe major attachment events, potential threat of separation, illness, solitude, death, and abuse. The stimuli are: child at window (window); departure; bench; bed; ambulance; cemetery; and child at corner (corner). Individuals are asked to make up a story for each image in which they describe what is going on in the picture, what led up to the scene, what the characters are thinking or feeling and what might happen next. The responses are audiotaped for transcription and verbatim analysis. The AAP assesses attachment in the Bowlby-Ainsworth tradition (West and George, 2002; George and West, 2012). The AAP Coding System, leads to four adult attachment classification patterns, – secure/autonomous, dismissing, preoccupied, unresolved – as they were traditionally assessed in the AAI, even if no multiple scoring is allowed. The AAP also assesses attachment personal elements that individuals may exclude from conscious awareness. Attachment classification using the AAP is determined by evaluating patterns of responses using a set of seven scales grouped under three major categories: discourse, content, and defensive processing. These dimensions evaluate the attachment story content related to the hypothetical characters portrayed in the stimuli, to defenses, and to self-other boundaries in narrative discourse (George and West, 2001, 2012). Discourse codes evaluate personal experience. Content codes include agency of self and connectedness for alone pictures, and Synchrony for dyadic pictures. Finally, the AAP codes for defensive exclusion, segregated systems, deactivation, and cognitive disconnection (Bowlby, 1980). They represent different degrees of “protection” from dangerous distressful events. Segregated systems describe a mental state in which painful attachment-related memories are isolated and blocked from conscious thought and rooted in experiences of trauma or loss through death (Bowlby, 1980). Deactivating defensive processes are defined as attempts to dismiss, cool off, or shift attention away from attachment events, individuals, or feelings in response to the picture stimuli. Cognitive disconnection processes literally disconnect the elements of attachment from their source, thus undermining consistency and the capability of holding in one’s mind a unitary view of events, emotions, and the individuals associated with them. The most recent review of AAP reliability and validity was published in George and West (2012). AAP–AAI convergence for secure versus insecure classifications was 0.95 (κ = 0.75, *p* = 0.000); convergence for the four major attachment groups was 0.89 (κ = 0.84, *p* = 0.000; George and West, 2001, 2012; West and George, 2002). The AAP has also been shown to be useful in studying the neurobiological and emotional expression correlates of attachment in non-clinical and clinical samples (Buchheim and Benecke, 2007; Buchheim et al., 2007, 2008, 2009; Fraedrich et al., 2010) as well as in single case studies (Lis et al., 2011).

Both AAI and AAP show individual strengths in measuring attachment patterns, but their combined use increments their overall usefulness. The AAI, the golden standard measure of adult attachment (Bakermans-Kranenburg and van Ijzendoorn, 1993), focuses on the assessment of the representational model and coherence of mind, and assumes developmental continuity of the attachment system, evaluating abuse and loss in one’s personal history. The AAP, based on the Bowlby–Ainsworth tradition (West and George, 2002; George and West, 2012), assesses current views of self, attachment figures, and expectations about the productiveness of attachment relationships, elucidating how current experience activates attachment accomplishment, disappointment, and trauma from the past (West et al., 1995; George and West, 2012). The AAP is also more trauma sensitive and underscores defense patterns (e.g., Hesse, 2008; George and West, 2012). The combined use of the AAI and the AAP gives the chance to portray a complex image of the patient’s attachment pattern, providing a detailed narrative about life attachment activators such as separation, fear, solitude, and danger, shedding light on the unconscious defensive mechanisms and exploring the accessibility of attachment figures during the life-span (e.g., Hesse, 2008; George and West, 2012). The SCL-90-R contributed to get Matilde’s self-evaluation of symptoms.

## ASSESSMENT FINDINGS

Results from DSM-IV diagnosis, SCL-90-R and SWAP–200 during the assessment phases are described in **Table 2**. Results from attachment tools are reported below and AAI subscales are shown in **Tables 3** and **4**.

**Table 2:** Results from SCL-90-R and SWAP–200 in assessment phase.

Diagnosis	Assessment
DSM-IV	Axis I: panic attack disorder
SCL–90-R	GSI = 1.14
	SOM = 1.20
	O–C = 1.40
	DEP = 1.85
	ANX = 1.70
SWAP–200	PD Factor
	Obsessive–compulsive (68)
	Schizoid (60)
	Q Factor Avoidant style (60.69)
	High Functioning (55.40)

*For descriptive purposes, we report only meaningful values of the different instruments, using SCL-90-R clinical cut off = 1.20, and SWAP–200 cut off of 50 < T ≤ 60 (some evidence) and T > 60 (high evidence).*

**Table 3:** AAI experience scales.

Experience scales		
	Mother	Father
*Loving*	7.0	5.5
*Neglect*	1.0	1.5
*Rejection*	1.5	1.5
*Pressure to achieve*	1.0	3.0
*Role Reversing*	1.0	1.0

*AAI State of Mind Scales end Experience Scales range from 1 to 9 (1–3 very low; 4–6 medium; 7–9 very high).*

**Table 4:** AAI state of mind scales.

	State of Mind			

	**Mother**	**Father**		
*Idealization*	2.5	2.0	*Lack of memory*	4.0
*Derogation*	2.0	3.0	*Preoccupied anger*	1.0
			*Passivity*	1.0
			*Fear of loss*	3.0
			*Metacognitive*	4.0
			*Coherency transcript*	6.0
			*Coherency mind*	6.0

*AAI State of Mind Scales end Experience Scales range from 1 to 9 (1–3 very low; 4–6 medium; 7–9 very high).*

Matilde’s AAI was scored F2/Ds3, secure with features of dismissing or some restriction in feelings of attachment (F2 = free somewhat dismissing or restricted in attachment; DS3 = dismissing restricted in feelings with some evidences of Lack of Memories; George and Solomon, 1996; see **Tables 3** and **4**). Matilde secure pattern was so defined because she was able to explore his or her thoughts and feelings about childhood experiences, with fresh speech, humor and forgiveness, without becoming angrily or passively overwhelmed while discussing them. Generally, Matilde appeared to be aware of the nature of experiences with her parents and of the effects of such experiences on her present state of mind and on her personality. Nevertheless, she remained a little bit restricted in her emotional expressions, preferring to rationalize. State of mind scales tapped a dismissing feature, showing a slight tendency to idealize parents and some lack of memories. Further information about Matilde derived from a qualitative analysis of the AAI. She did not report any severe illness, traumatic or abuse experience. However, separations caused her some distress, but she felt always supported and listened by her mother. She described herself as a very calm girl but, during early childhood, she was very shy and very worried about separation: “*I became very agitated when I did not see my parents, when they were not there, when they were away from home,*” “*Once we were at the lake. I was on the one side of the road and my parents were on the other side. Some people passed and so I could not see my parents anymore. I did not see them anymore and I began to scream*.” However, she remembered that during her summer camp experience, when there were no well-known friends or schoolmates: “*I felt the distance from home, I felt lost and confused … I was very happy to go back home. The bus journey to go back home was very stressful,” “I do not like changes. I am worried about changes.*”

She described the relationship with her mother as: affectionate, playful, reciprocal, supportive, and protective. When she was asked to recall a specific example in respect with “supportive relationship” she reported that “*I gulped a small toy and it remained caught in my throat. I had to be taken to the hospital. I was very agitated, I screamed that I was frightened of dying. Mammy was very supporting … I mean comforting.*” She was able to identify a specific episode, but in a superficial and not qualitative consistent manner (*Grice’s qualitative* *maxim*): the adjective-descriptor (supportive) of relationship with her mother was supported with a second generalized positive descriptor (comforting). The adjectives she chose to describe the relationship with her father were: always affectionate, playful, formal (*“home rules had to be respected, for instance times for lunch and dinner”*), respectful (*“Nothing escaped from him; his words had always a weight”*), and important. Such aspects were more linked to father’s role as a parent and to school achievement:* “I felt very bad about his criticism.”* Matilde felt closer to her mother than to her father, from whom she felt more detached. Moreover, during school years she always felt a little bit anxious and agitated about school achievement and completion. Beside all these difficulties, she always felt supported and sustained by her mother, and at the end she demonstrated herself as very forgiving toward her father’s severity. When asked to imagine the possibility of being separated from her child, Matilde reported to feel “*a big void, a big feeling of lost, of mourning, a big pain, an absence of being complete*” and the three wishes about this child when he would be 20-years old were: “*to be able to choose, to have a clear reasoning, not being confused, and to be able to be autonomous.*”

Matilde was judged as secure on the AAP (F): she showed, at the representational level, a flexible and organized thinking about attachment situations and relationships (Bowlby, 1969/1982). She was confident that she could rely on attachment figures to achieve care, safety and protection and, when alone, she could access internalized attachment relationships (George and Solomon, 1996, 1999). In response to two of the alone stimuli – Window and Cemetery- as a secure individual, she demonstrated the ability to think (i.e., Internalized Secure Base) and to take constructive action. She also used flexible defensive processes to integrate attachment feelings and events. Using these resources she was able to re-organize her attachment-related feelings, also in the few cases (Bed and Cemetery Stories) when she became disorganized by feelings of loss and danger. From the pattern of story responses it appears that Matilde, above all other response qualities, genuinely valued and represented the capacity for integration of self and relationships. The responses to the Alone pictures demonstrated Matilde’s internal resources, such as the potential availability and responsiveness of attachment figures. As a representative example of this attitude the *Cemetery* picture (a man stands by a gravesite headstone) story is reported.

“A gentleman who had a bad day or felt sad or depressed or undervalued because of an episode that happened during the day and goes and visits his father … he feels reassured because he found a place where to think about his life by himself and then he will go back home and will be able to reconsider what happened from a different point of view.”

In Cemetery, Matilde reveals the intensity of feelings of pain associated with loss: she tries to deal with them through some form of uncertainty and desire to withdraw (cognitive disconnection). These forms of organized defensive mechanisms keep Matilde’s attachment system activated but they cannot prevent her from becoming dysregulated, as evidenced by painful attachment-related feelings of loss represented by the appearance of a them where no clear distinction is made between life and death (“he goes and visit his father”). A segregated system (spectral domain) was activated by the picture features, which portray a man visiting a grave. However, Matilde was able to depict the man as engaged in some kind of “thinking.” The man is able to “reconsider what happened from a different point of view.” This process belongs to Internalized Secure Base, and portrays Matilde’s ability to clearly differentiate between the living and the dead. The Dyadic picture stimuli portray attachment-caregiving dyads. The responses to Dyadic picture stimuli demonstrate Matilde’s representation of the self and other in attachment situations when attachment figures are present and accessible, but they also demonstrate the use of attachment figures to quell the attachment anxiety aroused in the scenes depicted in the cards. Bed picture (a child and woman sit opposite to each other on the child’s bed) could be a representative example.

“A boy had a nightmare during the night and his mother woke up eh … now he is scared and he would like to be close to his mum … the mum is trying to soothe him and she will be able to do it … the boy will come back to sleep quietly … (Anything else?) no.. maybe the bad dream was … was about the fact of staying alone without his mom … and now … he wants his mom first!”

In Matilde’s story, the child signals his attachment need after a “nightmare” (segregated system in AAP) and the mother is able to provide a contingent and soothing answer, containing the potential breakdown of the attachment system and resolving the segregated system. Both AAI and AAP classified Matilde as secure with somewhat dismissing or restricted feelings in attachment without elements of unresolved abuse or trauma. However, both tools detected some shortcomings about fears of separation and danger. The AAI was not able to draw attention in an exhaustive way to how Matilde experienced abandonment fears and felt scared without the presence of her parents. Instead the AAP clearly depicted this nuance, under a secure pattern, showing that her attachment was threatened by painful attachment-related feelings of loss, and by a nightmare (in Bed picture), a signal of danger. In both tools she demonstrated her ability to re-organize herself, but these disturbing feelings kept being alive underneath her reorganized secure pattern.

### CASE FORMULATION BASED ON PDM AXES

*S*-axis – Matilde had a diagnosis of DSM-IV (American Psychiatric Association [APA], 1994), and showed a slightly High Functioning profile with Obsessive, Schizoid-Avoidant and Dysphoric characteristics, in both PD and Q factors in SWAP–200. Matilde’s SCL-90-R symptom profile revealed depression, anxiety, obsessive–compulsive, and somatization scores in the clinical range (**Table 2**).

*M*-axis – this Axis describes nine dimensions, which systematize the capacities that contribute to an individual’s personality and overall level of psychological health or pathology.

#### Capacity for regulation, attention, and learning

In the clinical interview, she said, “*I lost control of my body and thinking.*” She appeared in a profound state of crisis and she appeared to be unable to cope with it and with connected feelings of anxiety and distress. She (a) adhered rigidly to daily routines and became anxious or uncomfortable when they were altered, (b) had trouble making decisions and was indecisive or vacillated when faced with choices, (c) was overly concerned with rules, procedures, order, organization, and schedules: all obsessive strategies which would interfere with processes that support attention and learning from experience. However, according to Bowlby, being secure at both AAI and AAP means that Matilde had basic capacities for regulation. Matilde appeared to believe in the seeking of proximity and support as effective ways in regulating distress, in particular in AAP Dyadic pictures. However, at the moment of assessment, she was not able to recur to her internalized security patterns, showing how an emotional regressive crisis was rising up. Although Matilde subjectively felt unable to cope with it and was very frightened by it, according to the AAP and AAI, the dysregulation appeared momentary and not prolonged. It seems she was still functioning as she described herself in the early childhood memory, when she could not see her parents and she got anxious at the thought of being lost. However, her basic secure attachment suggests that, thanks to the therapy, she could re-establish her capacity of self-regulation, a secure person’s basic characteristic.

#### Capacity for interpersonal relationships

Although she was excessively devoted to work and productivity, compromising leisure and relationships, her secure attachment pattern at the AAI and AAP indicated that Matilde had a positive representation of available adults who can offer protection, support, care, and comfort in threatening and stressful situations. The AAP supported also her potential ability to be connected with other relational systems such as partners and peers, almost in a concrete manner, since she was able to tell stories in which she described specific connections with friends and other people in general. However, her agency, connectedness, and synchrony were at the moment “quite silent” in her everyday life. She needed help to regain these resources.

#### Quality of internal experiences

In AAI and AAP she felt reassured by (her) mother’s proximity, soothing, and comfort. However, episode and story plots clearly indicated some separation anxieties and worries in respect to changes, which she faced using dismissing defense mechanisms. AAP clearly depicted how under a secure pattern, her attachment was threatened by painful attachment-related feelings of loss and danger. Until that moment, such feelings were isolated and blocked from conscious thought. Even if she was able to deal with these experiences in childhood thanks to her mother’s comfort, her fear of loss connected to the fear of being alone and unprotected seem to re-emerge We hypothesized that she was having trouble in coping with new adolescence-through-young adulthood tasks, such as the adult separation-individuation process. According to the SWAP–200, she experienced a sense of personal dissatisfaction, poor self-regard, low self-esteem, lack of confidence, and chronic self-criticism. Her unrealistically high standards together with her expectation of being “perfect” above all in her achievements, made her feel guilty, depressed and despondent, with negative self-regard toward others, and the world at large.

#### Affective experience, expression, and communication

According to the SWAP–200, Matilde tended to defend herself via the inhibition of emotion expression, by means of abstract thinking and intellectualized terms, and appeared unable to recognize her wishes and impulses. Apparently, intellectualization and disavowal defenses (above all rationalization) led her to the avoidance of expressed conflict and emotions – both positive and painful. This emotional constriction resulted in a bottled up affect being channeled into panic attacks. The SWAP–200 stressed the risk of recurrent episodes of overt anxiety, tension, nervousness, and irritability and difficulty in acknowledging or expressing underlying feelings of anger and resentment. The AAP and AAI confirmed that underneath this block of affection there was a rich and positive affective state she had internalized during childhood life experiences. However, although not so rigid, her present affective state of constriction and inhibition was consistent with the rigid attempt to neutralize affect using deactivating defenses in the AAP. The emotions, which were bottled up in the segregated system, surely carried a negative and overwhelming emotional tone, which at the moment she was unable to deal with.

#### Defensive patterns and capacities

The SWAP–200 indicated the extent to which Matilde tended to defend her from expressing emotions, by abstract thinking and intellectualized terms. Although her defenses were at a mature-neurotic level, they were not solid enough to allow her to avoid the recourse to symptoms and anxiety. From an attachment viewpoint-AAP-Matilde shows a different picture underneath. Here defenses appeared organized and flexible, but in order to keep a regulated attachment she relied more on deactivation than on cognitive disconnection (George and West, 2012).

#### Capacity to form internal representations

Matilde was able to form internal representations of self and others, and her experiences were symbolized mentally. However, in the current state of distress, some emotions and conflicts were expressed somatically through her somatic symptoms and panic attack episodes.

Capacity for differentiation and integration (ego strength, self-cohesion, stability of reality testing). Overall, her AAP stories and her narrative in the AAI revealed that she was able to look realistically at herself, people, and relationships. Also during the clinical interview, a solid, stable and good child image emerged, but was not integrated with an adolescent and adult image: Matilde’s ego was fragile and was shattered by a large number of symptoms, her self-image was damaged and not well integrated; moreover, she looked younger than her age and never talked about sexuality or intimate relationships.

#### Self-observing capacities

Matilde did not demonstrate good self-observation capacities. Her level of current distress, extension of intellectualization and rationalization defenses, avoidant and constricted emotional style did not allow for an adult and mature emotional insight.

Capacity to construct or use internal standards and ideals. SWAP–200 showed how she currently set unrealistically and childish high standards for herself and how she appeared intolerant of her own human defects.

## THERAPEUTIC STANCE AND THERAPY GUIDING CONCEPTION

Matilde looked younger than her age and did not talk about sexuality or intimate relationships and we supposed that she did not undergo a true adolescent process. Looking at her secure attachment pattern, the therapist hypothesized that the present state of dysregulation and symptomatic picture is transitory and derived from the new young adulthood tasks she has now to deal with during her transition toward adulthood, such as moving to University. From a psychoanalytic as well as an attachment-oriented viewpoint, we hypothesized she was not able to face adolescent and adult separation-individuation processes. According to attachment theory, attachment relationships foster integration of attachment with relationships in peer behavioral systems during adolescence and adulthood: these include friendships and romantic relationships (West and George, 1999; Allen, 2008; George and Solomon, 2008; George and West, 2012). Psychoanalytic theories also agree that the individual needs to face adolescence as a separation-individuation process, where adolescents need to acquire an individual separate self-identity through identification with parents and separation from childhood ties. The AAP and AAI were taken into account, making the therapist sensible to specific topics concerning separation, loss, and loneliness, able to reactivate and unleash childhood attachment-related memories of fear of being lost and completely alone during treatment itself. Matilde needed to explore this topic with a therapist who would represent for her, in the transference, a secure parent similar to the one she had already experienced in her life via her mother’s supporting stance. In particular, the therapist expected that supportive psychotherapy would integrate the “segregated” themes locked in Matilde’s experience: she might then be able to consciously accept and deal with her blocked emotions and affects, as to regainenjoyment of life and satisfaction in the relationship with significant figures, re-finding the *haven of safety* of self and others that she had experienced in her childhood.

The clinical case formulation suggested for Matilde a therapeutic approach in the context of a “partial rapprochement” between attachment theory and psychoanalytic individual psychotherapies as the best solution (Skean, 2005; Slade, 2008; Steele et al., 2009). This intervention would include: (a) The use of therapeutic relationship and alliance as vehicles for a “secure base” constitution, in order to observe and understand the client’s interpersonal behavior (Spence, 1982; Binder et al., 1987; Dozier et al., 1994; Slade, 2008; Steele et al., 2009); (b) Relationships with the self and others (internal and external), in terms of personality functioning but also from client’s transference and therapist’s counter-transference points of view of (McWilliams, 1999; Skean, 2005); moreover, patient’s real or transferential relationships and past-present pattern of emotional responses and behaviors were examined (Gabbard, 2009). However, a particular emphasis was put on the supportive versus insight-oriented modes of therapy (Skean, 2005), because Matilde needed: (a) to reduce physical and psychical symptoms (*S-Axis*; Gabbard, 2009), and reestablish a consistent level of functioning (Dewald, 1971; Ursano and Silberman, 1996; Douglas, 2008); (b) to strengthen her fragile ego. Her defenses were at a mature-neurotic level, but were not solid enough to stop the recourse to symptoms and anxiety (*PDM: Defenses;* *Capacity for Differentiation and Integration*), (c) to change her self-definition, improving self-esteem, and getting a more integrated perception of the self, (*PDM: Qualiy of internal experiences; Capacity for Differentiation and Integration*); (d) To function better in everyday life investing lessin achievements and study matters (Dewald, 1971; Ursano and Silberman, 1996; *PDM: Rehabilitation*); (e) to improve her coping skills and to learn consistent strategies to manage her painful internalized feelings (*PDM: Capacity for regulation, attention, and learning*); (f) to increase her capacity to express affects both on the positive and negative aspects evidenced by AAI and that were consciously often inhibited and not acknowledged *(PDM: Affective Experience, Expression, and Communication)*; (g) to encourage more consistent ways of relating to others (*PDM: Capacity for Intepersonal Relationships;* Misch, 2000). In addition, her concrete and intellectualized thinking (SWAP–200) made also difficult for her to deal with interpretations, suggesting again the need of a more supportive approach.

### BRIEF OUTLINE OF THE THERAPY UNFOLDING

As expected, during the first months of therapy Matilde showed a high symptomatic picture. She appeared very distressed and confused, with a sense of failure, of inability to reach her standards. Long boring and intellectualized descriptions of daily routines and of University achievement, anxiety, uncertainties and doubts about her achievements were her main topics. The therapist acted as a secure attachment figure (Parish and Eagle, 2003; Mallinckrodt et al., 2005), as a caregiver who offered security and soothing to Matilde’s distress. She worked actively helping Matilde to contain anxiety, shame, and anger (Winston et al., 2004). The therapist, very slowly and respecting her defenses, tried to reduce Matilde’s anxiety, to increase her self-esteem and hope, and to make her more aware about herself as a person, and not only as a student who had to achieve some standards. She begun anyway to talk about how she could count on her mother, the only person that always helped her when she felt anxious and distressed. This finally opened a window on her family and her separation difficulties during childhood, and she started to tellhow she felt alone and how much she needed her mother’s soothing, how much difficult it was to face her first experience of a 2-week summer camp, as well as to begin elementary school, middle school, and high school. She also admitted that anyway with her mother’s help she was able to face these separations. She began to recognize, following therapist’s verbalizations, that at that time she was beginning a new kind of “school-experience,” similarly to the situation at present. In parallel with this initial understanding of her fear of facing changes and separation, all symptoms increased, especially anxiety symptoms. “*It is a nightmare*” were her words. She told the therapist that she called mommy every morning and evening but it was not enough. She felt lost and alone. The episodes reported by Matilde at this phase of the therapy were very similar to the ones she reported in the AAI, and the ways she dealt with the present separation from her mother were similar to the ones she previously used during her childhood: going concretely to her mother to be soothed and supported. Moreover, she used the same words she previously used in the two AAP stories where segregated systems were unleashed but resolved. It could be hypothesized that in the transference with the therapist Matilde’s attachment system was activated and “seen in action” (George and West, 2012). As she said during the AAI, it was always difficult for her to deal with changes. Now in the transference with the therapist she was reliving her fears, the same fears she experiencedin childhood, the ones that were unleashed at the beginning of the University andthat she was able to face only through anxiety and obsessive symptoms. It was difficult for her to connect this experience with the new separation experience from home and from herself as a child, now that she had to face University and all the complex processes connected with entering adulthood. In the transference with the therapist she was reviving an acute separation anxiety and she was also unconsciously angry at the therapist’s impossibility to help her. The therapist tried unsuccessfully to interpret and to connect this profound regression with Matilde’s previous separation anxieties. Words were not useful. Wallin (2007) supports that “*what patients are unable to explain with words, tends to be evocated, enacted or incorporated*” (Zaccagnini and Zavattini, 2009). The working alliance showed for the first time some ruptures, and the risk of treatment disruption itself became a subject of discussion (Appelbaum, 2005; Colli and Lingiardi, 2009). Matilde’s alliance rupture style was characterized by the presence of withdrawal maneuvers: emotional disengagement from the therapist, skipping from topic to topic, responding in an overly intellectualized fashion, and very short answers (Safran and Muran, 2000). In such a moment of regression, she really needed a concrete comfort and physical contact with her mother. The therapeutic stance was not enough for her; she decided that the best way to deal with the situation was to go back home. Matilde went home, “*to be near to her family.*” Coming back to her parents represented for her the *haven of safety* she described in her AAP. Parents were still used as attachment figures during early, middle and late adolescence and also during young adulthood (Fraley and Davis, 1997), especially under conditions of extreme stress (Huntsinger and Luecken, 2004; Kamkar et al., 2012). She stayed home with her family for 3 weeks. When she came back, she appeared less anxious and more integrated: little by little, Matilde was more able to feel the setting as a place where exploration of her personal life and new experiences could be initiated, shared, and enjoyed. She was able to develop positive feelings toward the therapist (Misch, 2000). She began to integrate positive and negative feelings in life events, becoming more and more flexible, increasing her ability to tolerate changes and learning to find new solutions to life schedule. She reached some goals toward adulthood and began to find real friends, also far from home, and to spend energy in different activities (e.g., organization, church, neighborhood, etc.). She loved challenges and she felt pleasure in realizing her goals and in pursuing long-term ambitions. A boyfriend appeared. The symptoms disappeared. She continued to use a great amount of razionalization in order to explain some affective aspect of her experiences. from the point of view of attachment, she mantained a tendency to change the topic when she approached emotional issues using displacement defenses in order not to deal with her core difficulties. The therapeutic goal of accomplishing a true adolescent process was also achieved. A solid and good child image was now more integrated with an adolescent and adult image. Some developmental tasks were reached on the way toward adultood (friendship and romantic relationship). The therapist discussed with Matilde the fact that some shortcomings were still present in her personality functioning, but both agreed that she wished now to try to go on with her life by herself. As Freud (1966) suggested, the aim of psychotherapy at a developmental age is to help the patient to proceed along his or her developmental lines. Now Matilde managed to integrate some issues concerning the developmental step of adolescence and young adulthood and she wished to try new experiences by herself.

## FOLLOW-UP FINDINGS

Results from DSM-IV diagnosis, SCL-90-R, and SWAP–200 in the follow-up phase are described in **Table 5**.

**Table 5:** Results from SCL-90-R and SWAP–200 in follow-up phase.

DIAGNOSIS	Follow up
DSM-IV	Axis I: No diagnosis
SCL– 90R	GSI = 0.69
	SOM = 1.10
	O–C = 1.10
	DEP = 0.77
	ANX = 1.10
SWAP–200	PD factor
	Obsessive–compulsive (62.82)
	Q factor
	Obsessive style (70.50)
	High functioning (66.25)

The AAP was scored secure, but without any segregated systems. As a representative example of some new attitudes, Matilde’s stories for *Window* and *Departure* pictures are reported.

Window (a child looks out a window): *a girl who woke up eh … parents are not there, they are at work and she knows she is alone at home. She is quiet. She is looking out of the window thinking about her mom and the fact she will come back home in the afternoon. She is thinking about who she can invite in … who can … can keep … her company. She is quiet, excited by the day without parents (What might happen next?) she will find someone … a … a friend … a neighbor … finally she will have fun (Anything else?) No.*

Matilde tells one of the most common AAP stories for the Window picture: a typical home-related scenario in which a little girl needs to manage her solitude. The girl is “*quiet*” although she is alone home. So, Matilde is not threatened by the girl’s loneliness, but she is somehow able to enjoy the possibility of being alone. The absence of segregated systems demonstrates the absence of dysregulating events, which could have led to her being alone (her parents are just working) and of girl’s traumatic reactions. More specifically, the girl is depicted “*thinking about her mom,*” activating her ability to internalize the secure base and being “*content in solitude.*” In fact, this connection with the thought of her mother’s coming back in the afternoon keeps the girl regulated and lets her also think about something specific to do alone: “*she looks out of the window thinking about who she can invite in … who can keep … her company.*” The little girl can recall the affiliative system (“*friend*”) to handle her loneliness. Her ability to think makes Matilde confident and envisages the possibility of changing things in the immediate future (“*she invited friends*”). From a developmental point of view, Matilde is now a late adolescent-young adult: she is prone to consider also peers and friends as a secure base to refer to in moderately distressful situations.

Departure (an adult man and woman stand facing each other with suitcases positioned nearby): *a woman is going to leave for a business trip and she is saying goodbye to her husband … he took her to the station … she was already planning what she needed to do during the trip … yes during this period of work … he is quiet and he thinks about their relationship, about how they enjoy to be together, what he would do without … in these few days without his wife…however … she will leave and he will spend a few dull days … (Anything else?) No.*

In Departure, Matilde is able to tell a typical AAP story, which portrays a couple at the train station. The husband thinks about their relationship and he feels that his days will be dull without his wife. Matilde’s story suggests togetherness and a goal-corrected partnership. She portrays the husband as involved in a contingent, reciprocal and mutually engaging relationship.

### QUALITATIVE CLINICAL EVALUATION AT THE END OF THERAPY

Matilde did not have any DSM-IV diagnosis in Axis I and her personality functioning resulted carachterized by obsessive high functioning features (PDM *S*-axes, SWAP–200). She had no more panic attacks accompanied by strong physical arousal, and her experiences were now more mentally symbolized. Her SCL-90-R final symptom profile revealed a magnitude within the normal range. Only two symptomatic distress levels, obsessive–compulsive, and anxiety, still penetrated the clinical range, but their intensity had diminished compared to the assessment phase.

Her self-image improved: she now experienced a sense of personal satisfaction, sufficient self-esteem and self-confidence (PDM *M*-axes). The “negative-stressful” components of her affective world were still present but the level of self-blame, and emotional constriction greatly diminished. She was now less inhibited and became more spontaneous in expressing emotions, and also anger. Matilde still showed a pattern of Mature-Neurotic defenses and an absence of primitive defenses. Rationalization, intellectualization, undoing, and displacement were the mostly used defenses, but were now more flexible, less pervasive and she was able to avoid recourring to symptoms and anxiety. The AAP confirmed a flexible use of defenses and reduction in thereliance on dectivation defenses; however, her kind of defense structure still did not allow neither an emotional insight about her motivations and behaviors, or psychological mindedness.

Matilde remained secure in her attachment pattern, changing her defensive approach through a more integrated and coherent one, in which no more segregated systems or disregulation were present: she was now able to use again her self-regulation capacities autonomously (PDM *M*-axes). Her attachment adolescent crisis was resolved; she was out of her “nightmare.” She still seemed naïve and used an excessive part of her mental energy to keep emotions and feelings at bay, showing a limited ability to appreciate metaphor, analogy, or nuance. In the context of a supportive stance, comprising a secure and holding environment and an atmosphere based on emotional safety, she was able to work on her fear of loss and changes allowing her internalized attachment status to reach an adult structure. She was now able to deal with adult tasks using her internalized parental figures, thinking about how they could protect her. Matilde now found pleasure, satisfaction, and enjoyment in everyday-life activities. Her underneath security pattern now re-emerged allowing her to maintain a loving relationship, and to engage and keep long-standing and intimate friendships and relationships (PDM *M*-axes). She now set less unrealistically high personal standards and she was now able to find meaning in belonging and contributing to a wider community (e.g., organization, church, neighborhood, etc.). **Table 6** shows Matilde’s qualitative picture at baseline and at follow up.

**Table 6:** Qualitative Matilde’s picture of features at baseline and at the end of psychotherapy.

	Baseline	Follow up
Symptoms and behaviors	Panic attacks episodes, anxiety and depression feelings, self blaming, self-closure to experiences, obsessive–compulsive approach to life and university study, general avoidance stile	Some self-blaming, obsessive–compulsive approach university study, high functioning and adjustment, openness to life events and relational experiences
General adjustment and life goals	Some difficulties in University exams	Better capacity in coping with university exams
	Unable to stay alone	Better capacity to stay alone and feel pleasure to daily life
	Unable to have significant or romantic relationship	Higher investment in social activities
	Unable to feel and found pleasure in daily life and activities	Construction of significant relationships with pairs and engagement in romantic relationship

## DISCUSSION

This clinical case study highlighted the importance of assessing patient’s idiographic and intra-subjective features (Hilliard, 1993). The nature of the clinical case perspective requires a rich diagnostic process that includes both a nosographic approach (such as DSM-IV) and a more multifaceted point of view to assess the specific patient’s psychological functioning (Barron, 1998; Shedler and Westen, 2007). There have been few studies investigating the psychotherapy process in supportive therapies (Orlinsky et al., 2004), and very few studies were devoted to inserting also the contribution of validated measures of attachment. Slade (2008) endorsed that, although attachment theory terms have been incorporated in the present psychoanalytic theory, only few therapists have really integrated core elements of the attachment perspective in their clinical thought. Above all, few of them inserted measures of attachment and their strategies to understand the therapy unfolding (Rockland, 1989; Porcerelli et al., 2011). Assessing attachment means more than just determining a patient’s attachment classification status. The benefit from the inclusion of attachment assessment to a multi-method approach is the chance of using results to elucidate the patient’s representational and defensive patterns related to attachment activation (Bowlby, 1980).

This paper tried to illustrate a clinical case where results from attachment tools together with PDM assessment could help to give a more integrate picture and to form and inform the unfolding of the therapy. The incremented validity about symptoms and attachment internal working models evaluation added a specific qualitative contribution to each tool (e.g., SCL-90-R gave the self perception of symptomatology and SWAP–200 the clinical perception of it; AAI and AAP increased biographical information and defense mechanism, respectively). The paper presented a case formulation in which a psychodynamic approach was integrated with an attachment theory framework both in the assessment and post-assessment phases and with a “*supportive psychotherapy approach.*” The secure attachment status, as derived from the AAI and the AAP, helped to structure Matilde’s therapy, adding information to the therapeutic intervention: Matilde’s secure attachment resulted helpful to establish a therapeutic plan, to facilitate the therapeutic alliance and the answer to the therapy, and to help her to face her symptoms and internal difficulties (Douglas, 2008; Steele and Steele, 2008). The AAP and AAI were taken into account, making the therapist sensible to the specific topics concerning separation and loss, which were reactivated throughout treatment. Matilde needed to explore them in the context of a “*safe haven,*” the same context she had previously experienced in her life with her mother’s supporting stance. On her side, the therapist recreated and maintained a well knownholding environment, affective mirroring and personal warmth (Markowitz, 2008) and an atmosphere based on emotional safety (Crits-Christoph and Connolly, 1999; Skean, 2005). She provided Matilde with the secure base she temporary lost, a starting point from where the exploration of painful experiences in her present life could finally begin. This gave her the possibility to recall some hidden memories, leading to self-exploration (Parish and Eagle, 2003; Mallinckrodt et al., 2005; Holmes, 2010). The “supportive approach” and the role of attachment framework turned out to be a key factor in the assessment and in the development of an effective therapeutic relationship with Matilde. Within a psychoanalytic framework, through the unfolding relationship with the therapist, Matilde brought her interpersonal world into the treatment room and allowed the therapist to experience aspects of her structuring of reality (Crits-Christoph and Connolly, 1999; Skean, 2005). The conclusion of the therapy showed a more integrated picture, where symptoms were no more outstanding and Matilde seemed to be out of her big “nightmare” and ready to face her life tasks in a more integrated and young-adult way. The post-treatment AAP confirmed that Matilde was able to integrate these issues of separation and loss. She was a very defensive neurotic patient blocked at latency, and showed some shortcomings related to the separation-individuation process (Mahler et al., 1975) both from a psychoanalytic and from an attachment point of view.

The treatment helped Matilde to make a developmental step toward maturity: “from childish features to adolescent ones, reaching the capacity of (emotionally) exploring the possibility of living independently from parents (…) because they know that they can turn to parents in case of real need” (Allen and Land, 1999, p. 322). The therapist was both an “attachment figure” that helped Matilde to face new experiences, as well as a transference object (Dozier et al., 1994). Matilde’s development resulted in increased abilities in managing the goal-corrected partnership with each parent, in which behavior is not determined only by adolescent’s current needs and wishes, but also by recognition of the need to manage certain set goals for the partnership (Bowlby, 1973).

As all clinical case studies, this study suffered from some limitations (Hodkinson and Hodkinson, 2001): results are not generalizable in the conventional sense; it looks expensive, if attempted on a large scale and the complexity examined is difficult to represent simply and briefly. Furthermore, clinical case studies results stronger when researchers’ expertise and intuition are maximized, but this raises doubts about their “objectivity”: this type of research is easily subjected to criticisms by those who do not like the messages that they contain; and finally it cannot answer a large number of relevant and appropriate research questions that future studies could address (e.g., in this sense, it could be highly valuable for future research to compare PAD patients with different attachment styles). However, this particular case study could be considered an original and extremely valuable one, because it is grounded in “lived reality.” This helps us to understand complex inter-relationships between diagnosis, measures and their clinical application, facilitating the development of conceptual/theoretical issues and the exploration of unexpected and unusual situations, such as PAD in a secure attached patient. As regards the choice of this patient, the present paper can provide “provisional truths, in a Popperian sense” (Hodkinson and Hodkinson, 2001): it represents the best account of such assessment and treatment in the current literature, and it should stand, until contradictory findings or better theories are developed.

## Conflict of Interest Statement

The authors declare that the research was conducted in the absence of any commercial or financial relationships that could be construed as a potential conflict of interest.

## Footnotes

1 The PDM was developed to describe “the depth as well as the surface of emotional, cognitive, and social patterns” (p. 1) of an individual’s functioning, as to improve the diagnosis and treatment of psychological disorders. PDM comprises three areas: personality patterns (Axis *P*), mental functioning (*M*-axis), and symptoms (*S*-axis). Our attention focused mainly on mental functioning or *M*-axis, “a microscopic look at mental life” (p. 8), although some attention was paid to symptoms and concerns or *S*-axis.

2 The patient self-referred to a psychodynamic service, where therapists are trained to use an Operationalized Psychodynamic Diagnosis approach during consultation sessions, preferring free or “per area” clinical sessions to interviews (e.g., SCID).

3 The self report SCL-90-R was digitally computed. Inter-reliability reached Cohen’s *k* = 1 for AAI and AAPs final classifications; 0.93 for AAI subscales; 0.85 for AAP codings; 0.72 for SWAP–200 final scales.

4 (1) Neutral (children playing ball); (2) child at window (alone); (3) departure (dyad); (4) bench (alone); (5) bed (dyad); (6) ambulance (dyad); (7) cemetery (alone); (8) child in corner (alone).
